# An Instance of Hypokalemic Periodic Paralysis in Adolescent Brothers: A Case Report

**DOI:** 10.7759/cureus.42082

**Published:** 2023-07-18

**Authors:** Ashley C Calise, Joel Carter, Tetiana Litvinchuk

**Affiliations:** 1 Medical School, North Alabama Medical Center, Florence, USA; 2 Medical School, Alabama College of Osteopathic Medicine, Dothan, USA; 3 Medical School, Hellen Keller Hospital, Sheffield, USA; 4 Pediatrics, Helen Keller Hospital, Sheffield, USA

**Keywords:** scn4a, cacna1s, flaccid paralysis, case report, hypokalemic periodic paralysis

## Abstract

Hypokalemic periodic paralysis (HypoPP) is a rare autosomal dominant disease caused by mutations in either calcium or sodium transmembrane voltage-gated ion channels of skeletal muscle or endoplasmic reticulum. Most cases of HypoPP are associated with a mutation in the gene encoding a calcium channel, the CACNA1S gene. Mutations in the channels create leakage currents that disrupt resting potential and depolarize the muscle fiber resulting in transient flaccid paralysis and low extracellular potassium (K+). Patients experience episodes of muscle paralysis typically provoked by exertion and diet. Treatment focuses on the prevention of such episodes with carbonic-anhydrase inhibitors or potassium-sparing diuretics as well as to treatment of acute episodes with oral K+ supplementation. Due to the rarity of the disease, the literature surrounding the disease and pharmacological management is limited. We present a case of two adolescent brothers who present with a confirmed diagnosis of periodic episodes of paralysis and are seeking treatment. Both brothers experience paralytic episodes provoked by acute changes in diet and exercise. However, the lack of literature and treatment guidelines surrounding the disease emphasizes the importance of documenting cases and the effectiveness of treatment outcomes. Additionally, it reminds providers to keep HypoPP on the differential when faced with a young patient experiencing paralytic episodes.

## Introduction

Primary periodic paralysis is a group of neuromuscular disorders that includes both hyperkalemic and hypokalemic periodic paralysis. Hypokalemic periodic paralysis, or HypoPP, is an autosomal dominant disease, meaning only one copy of the abnormal gene must be inherited to cause the disease, with an estimated global incidence of one per 100,000 [1]. The disease is typically caused by mutations in either calcium or sodium transmembrane voltage-gated ion channels of skeletal muscle or endoplasmic reticulum [2]. Approximately 60% of cases of HypoPP are associated with a mutation in the gene encoding a calcium channel, the CACNA1S gene, while 20% are associated with a mutation in the SCN4A sodium channel gene [1]. The abnormal CACNA1S channel is comprised of a missense mutation substituting arginine for histamine at 1q32.1 [3]. The American College of Medical Genetics and Genomics (ACMG) developed guidance for the interpretation of sequence variants and the CACNA1S gene is categorized as a pathogenic mutation due to its alterations of the electrophysiology of ion channels and potassium-dependent depolarization [3]. Mutations in the channels create leakage currents, resulting in an inward non-selective cation leak current that disrupts the resting potential and depolarizes the muscle fiber. Transient membrane depolarization causes inactivated voltage-gated sodium and calcium channels resulting in flaccid paralysis [2]. The disease is characterized by muscle fiber inexcitability with the presence of low extracellular potassium (K+) [1]. 

On disease presentation, patients experience intermittent episodes of muscle paralysis that can last minutes to days. Episodes of HypoPP typically last more than two hours and predominately affect the lower limbs [1]. The onset of the disease commonly occurs in the first or second decade of life with an average age of diagnosis of 15; Incidence is three to four times more common in males due to a higher penetrance of the gene for unknown reasons [4,5]. Attacks are typically precipitated by behavioral triggers such as diet, alcohol, and exercise and decrease in frequency with age. Such episodes are accompanied by changes in serum K+ level, often less than 2.5 mEq/L (reference ranges 3.5 to 5.1 mEq/L) [1].

Diagnosis is made clinically, by genetic testing for sodium, potassium, or calcium channel mutations, and by the presence of low serum potassium during attacks [1]. Due to the rarity of the disease, treatment options are limited. First-line treatment is a behavioral modification, such as avoidance of triggers; patients are advised to adhere to a low sodium/carbohydrate diet and to avoid dehydration [1]. Additional treatment includes K+ supplementation, carbonic anhydrase inhibitors (CAIs), and K+-sparing diuretics. Although the exact mechanism is unclear, CAIs work by promoting K+ diuresis and inducing a non-anion gap acidosis by increasing urinary bicarbonate excretion thus reducing the susceptibility to paralytic episodes [1] However, patients with SCN4A mutations are less responsive to CAIs, and using these can worsen symptom severity [1]. In the case of K+- sparing diuretics, overall serum potassium is increased resulting in the prevention of episodes; eplerenone is preferred to spironolactone due to spironolactone's anti-androgenic effects [1].

The lack of disease prevalence precipitates a lack of literature regarding the disease. As a consequence, clinical management guidelines and treatment options are severely limited [3].

Here, we present the cases of two adolescent brothers, 14 and 16 years old, who presented to their pediatrician with a diagnosis of CACNA1S gene mutation HypoPP seeking to establish care and obtain treatment for their disease. Both patients responded well to treatment with preventative K+-sparing diuretics and oral K+ supplementation to use during episodes.

## Case presentation

Two male brothers, 14 years old and 16 years old, accompanied by their mother presented to the pediatric clinic to establish care. They requested to receive potassium (K+) supplements for hypokalemic periodic paralysis. Upon further questioning, both boys were currently feeling healthy and had experienced minimal paralytic episodes. Since early childhood, the boys had episodes of lower limb paralysis and weakness, usually correlating to periods of physical activity. Their mother reported that she had an extensive family history of HypoPP; she reported the disease was present in her father, three paternal uncles, and two cousins- one male and one female. Previously, she had been treating the children with her own pottasium supplements to alleviate their symptoms. The 16-year-old was currently playing sports and needed the potassium to take before and after exercise; he reported manageable episodes during exertion that could be both prevented and treated with K+ supplementation. Over the years, he has managed his episodes with diet and appropriate K+ replenishment. The 14-year-old needed the potassium to take daily prophylactically, as his paralytic episodes were more frequent. He reported that his episodes were exacerbated by his diet which consisted of high-calorie snacks and sodas. Neither of the patients had a significant past medical history, no known allergies, and were not taking any prescription medications. Physical exam was within normal limits on both boys and no abnormal findings were found.

Prior to being seen in our clinic, the two brothers had received genetic testing at Vanderbilt University Medical Center to verify if they had hypokalemic periodic paralysis, and the records of genetic testing were obtained. The only other member of the family tested was their mother; all three individuals were positive for the CACNA1S gene, which is associated with autosomal dominant hypokalemic periodic paralysis and malignant hyperthermia. All three individuals were heterozygous for the gene mutation. Upon verifying that both boys had the disease they immediately sought a pediatrician to manage their disease and to obtain potassium supplementation as treatment. Their mother reported she does not use potassium or any other medications for her disease as she simply uses a low-carb, low-sugar diet. She encourages the boys to eat the same diet but reports it is more difficult for the younger of the two which, in turn, leads to more frequent episodes of HypoPP attacks for him.

During their visit to the clinic, blood was drawn to get a baseline measurement of their electrolytes, and pharmacotherapy options were discussed. The boys agreed to start a new medication, Dichlorphenamide 50 mg BID - a carbonic anhydrase inhibitor, which they had never tried in the past, as prophylaxis for their episodes. Additionally, potassium chloride 20 meq daily and as needed were prescribed. Upon follow-up and review of their lab values, both boys had normal levels of sodium, potassium, and chloride at the time of testing. All other lab values were within normal limits. However, due to insurance restrictions, dichlorphenamide was not initially covered and thus had not been started. So, the medication was switched to acetazolamide for use in the interim. The younger brother initially reported that potassium supplements were keeping paralytic episodes manageable as he continued to have episodes weekly when he consumed too much sugar or a high amount of carbohydrates in his diet. The older brother was continuing to use a potassium tablet before and after sports or exercise; he was also moderately controlling his symptoms through a modified diet. However, after some time on the new medication, the younger brother developed severe fatigue and increasing tiredness while taking acetazolamide with increased hypokalemic episodes, now happening two to three times weekly, and increased recovery time. Thus, the medication was stopped and repeat labs were normal. The boy’s parents declined to start another medication so we are awaiting insurance approval for dichlorphenamide.

## Discussion

HypoPP is a rare disease that can drastically affect a patient’s quality of life. Genetic analysis and low serum potassium, as well as clinical suspicion, remain the cornerstones of diagnosis. Patients experience flaccid muscle paralysis triggered, commonly, by exertion and diet. Treatment options are limited but typically consist of treatment for disease prevention with carbonic-anhydrase inhibitors or K+-sparing diuretics in addition to treatment for acute episodes with oral K+ supplementation. However, the literature surrounding the disease and appropriate pharmacological management is limited. This case, of two adolescent brothers who present with periodic episodes of paralysis, emphasizes the importance of documenting cases and the effectiveness of treatment outcomes. Additionally, it reminds providers to keep HypoPP on the differential when faced with a young patient who presents with periodic episodes of paralysis.

In a review of 40 cases of HypoPP, the prevalent mutations causing the disease were the CACNA1S and SCN4A mutations of calcium and sodium channels, respectively. CACNA1S mutations were present in 60% of cases [4]. Patients possessing this mutation also presented with lower serum potassium levels. The average age of diagnosis was 15.3 years ± 9.7 years [4]. Patients were treated with the carbonic-anhydrase inhibitor, acetazolamide; of which, only 50% of subjects responded to this treatment [4]. Patients reported better symptomatic management when treated with eplerenone. In another case report, a 14-year-old male presented with flaccid paralysis associated with hypokalemia. He reported two previous episodes a couple of years prior. His symptoms resolved after correcting serum K+ levels and the diagnosis of HypoPP was made [6]. 

Limitations exist in the management of HypoPP as the mechanism behind the disease is poorly understood. There remains a need for additional research in the management of HypoPP. The effect of treatment on preventing permanent muscle weakness or treatment impact based on specific genetic mutations is yet to be determined [7]. Additionally, HypoPP should remain on the differential of physicians when presented with episodic muscle paralysis as symptoms resolve rapidly with treatment [6]. The biggest challenge in the diagnosis of HypoPP is the recognition of the disease. When clinical suspicion is high, genetic testing can confirm the diagnosis [6]. Additionally, due to the autosomal dominant nature of the disease, most affected individuals have one affected parent. If a patient with the disease is considering pregnancy, prenatal genetic counseling can be considered. 

## Conclusions

In conclusion, when physicians are presented with a patient who exhibits episodic and variable paralytic attacks, hypokalemic periodic paralysis should be considered as a diagnosis. Detailed family history of muscular disorders should be obtained due to the autosomal dominant nature of the disease. Although treatment options are limited, current therapies can drastically improve the quality of life for patients with hypokalemic periodic paralysis by reducing the frequency of episodes and severity of symptoms.
